# Free energy estimation of short DNA duplex hybridizations

**DOI:** 10.1186/1471-2105-11-105

**Published:** 2010-02-24

**Authors:** Dan Tulpan, Mirela Andronescu, Serge Leger

**Affiliations:** 1 National Research Council of Canada, Institute of Information Technology, 100 des Aboiteaux Street, Suite 1100, Moncton, NB, E1A 7R1, Canada; 2 Department of Genome Sciences, University of Washington, 1705 NE Pacific St, Seattle, WA 98195-5065, USA

## Abstract

**Background:**

Estimation of DNA duplex hybridization free energy is widely used for predicting cross-hybridizations in DNA computing and microarray experiments. A number of software programs based on different methods and parametrizations are available for the theoretical estimation of duplex free energies. However, significant differences in free energy values are sometimes observed among estimations obtained with various methods, thus being difficult to decide what value is the accurate one.

**Results:**

We present in this study a quantitative comparison of the similarities and differences among four published DNA/DNA duplex free energy calculation methods and an extended Nearest-Neighbour Model for perfect matches based on triplet interactions. The comparison was performed on a benchmark data set with 695 pairs of short oligos that we collected and manually curated from 29 publications. Sequence lengths range from 4 to 30 nucleotides and span a large GC-content percentage range. For perfect matches, we propose an extension of the Nearest-Neighbour Model that matches or exceeds the performance of the existing ones, both in terms of correlations and root mean squared errors. The proposed model was trained on experimental data with temperature, sodium and sequence concentration characteristics that span a wide range of values, thus conferring the model a higher power of generalization when used for free energy estimations of DNA duplexes under non-standard experimental conditions.

**Conclusions:**

Based on our preliminary results, we conclude that no statistically significant differences exist among free energy approximations obtained with 4 publicly available and widely used programs, when benchmarked against a collection of 695 pairs of short oligos collected and curated by the authors of this work based on 29 publications. The extended Nearest-Neighbour Model based on triplet interactions presented in this work is capable of performing accurate estimations of free energies for perfect match duplexes under both standard and non-standard experimental conditions and may serve as a baseline for further developments in this area of research.

## Background

Predicting the stability of a DNA duplex from base sequences is a well studied problem nowadays. Nevertheless, the accuracy of DNA duplex stability predictions largely varies with sequence length, base composition and experimental conditions. The Thermodynamic Nearest-Neighbour (TNN) Model [1] is a state-of-the-art approach that is used to estimate the stability of a single or a pair of DNA (or RNA) molecules based on pairwise base interactions and structural conformations. A large collection of thermodynamic nearest-neighbour parameters were acquired by interpolation of results obtained from various experimental processes like NMR [2] and optical melting studies [1,3]. The accuracy of computing free energies for DNA duplexes is an important aspect for all prediction methods, considering their direct application for selecting, for example, microarray probes that perfectly hybridize with their complements within a pre-specified hybridization interval, while avoiding self-hybridization for each probe [4]. Here we select four widely used, publicly available computer programs that implement the TNN Model using large numbers of experimentally derived thermodynamic parameters, namely: the MultiRNAFold v2.0 package [5,6] with two sets of thermodynamic parameters, the Vienna Package v1.8.1 [7] and the UNAFold v3.5 package [8].

The MultiRNAFold package (including the PairFold program for duplexes) predicts the minimum free energy, suboptimal secondary structures and free energy changes of one, two, or several interacting nucleic acid sequences. The thermodynamic model for the thermodynamic stability of a joint secondary structure for two DNA or RNA molecules at a given temperature is performed similarly to that of a single molecule [9], except that an intermolecular initiation penalty is added. The PairFold algorithm uses dynamic programming to calculate minimum free energy secondary structures and runs in time cubic in the lengths of the input sequences (Θ(*n*^3^)). PairFold uses RNA thermodynamic parameters from the Turner Laboratory [10] and DNA thermodynamic parameters from the Mathews and SantaLucia laboratories [11,12].

The Vienna Package consists of a suite of computer programs and libraries for prediction of RNA and DNA secondary structures. Nucleic acid secondary structure prediction is done via free energy minimization using three dynamic programming algorithms for structure prediction: the minimum free energy algorithm of [13], which produces a single optimal structure, the partition function algorithm of [14], which calculates base pair probabilities in a thermodynamic ensemble, and the suboptimal folding algorithm of [15], which generates all suboptimal structures within a given energy range of the optimal energy.

UNAFold, the acronym for "Unified Nucleic Acid Folding", is a software package for RNA and DNA folding and hybridization prediction. UNAFold folds single-stranded RNA or DNA, or two single DNA or RNA strands, by computing partition functions for various states of hybridization. The partition functions will then help to derive base pair probabilities and stochastic samples of foldings or hybridizations. The package provides various energy minimization methods, which compute minimum free energy hybridizations and suboptimal foldings.

All three packages use similar dynamic programming algorithms for prediction of minimum free energy (MFE) and suboptimal structures and for partition function calculations. For the purposes of our work (i.e., DNA duplex MFE secondary structure prediction and free energy of hybridization), the main differences lie in the thermodynamic parameters used (SantaLucia or Mathews), and in the features considered (for example, the Vienna Package does not consider special types of poly-C hairpin loops in their model, whereas the other two packages do). Thus our **first goal **is to quantify the impact of these differences on the accuracy of DNA duplex free energy approximations. Throughout the paper, we use a set of measures that reflect the degree of similarity of calculated and experimental secondary structures and free energies. Based on these measures we quantify the accuracy of the predictions of the aforementioned programs using a collection of 695 experimental DNA duplex data that we collected from 29 publications.

We also introduce in this work an extended Nearest-Neighbour Model for perfect matches based on triplet interactions, that can approximate free energies for DNA duplexes under a wide range of temperatures, sodium and sequence concentrations. The model is similar to the one introduced in 1999 by Owczarzy et al. [16], the main difference residing in the inclusion of only triplet interactions for our model, rather than a mixture of singlets, doublets and triplets for the other. Thus, our **second goal **is to show that such a model outperforms simpler models based on doublet interactions and produces more accurate free energy approximations for DNA duplex hybridizations occurring in non-standard experimental environments (for example for different sodium concentrations or at different temperatures).

## Results and Discussion

In this work, we compare similarities and correlations of free energy values calculated using three publicly available packages, namely MultiRNAFold, UNAFold and Vienna Package and a Nearest-Neighbour (NN) Model for perfect matches based on triplet interactions. For this purpose, we collected and used a data set with 695 pairs of short DNA sequences and we investigated what method produces the closest value to the experimental free energy and under what circumstances. We acknowledge the fact that not all sequence lengths are equally represented in the benchmark data set simply due to their availability and thus our analysis may apply better to shorter sequences. The majority (91.37%) of experimental free energy calculations were obtained for perfect (0 mismatches) and almost perfect matches (1 mismatch), thus the current DNA parameter sets tend to have higher accuracy for close-to perfect match DNA duplexes. Another bias in the analysis may come from the fact that some authors have already tried to reconcile the existing differences in free energy model parameters [17,18] by optimizing sets of DNA parameters using the same sequences already present in the benchmark data set.

### Comparison of absolute differences between experimental and estimated free energies (MFE_AD)

We begin the presentation of our results by introducing a measure that provides insights into "worst" and "best" estimates for minimum free energies. Thus, the first comparison involves the absolute differences between experimental and estimated free energies (MFE_AD) among all the methods for model evaluation (column 3 in Table 1) and model prediction (column 3 in Table 2). In an ideal scenario, the estimated free energy would equal the experimentally inferred one, nevertheless in practice we would settle for a low absolute difference. In both scenarios, namely the evaluation of free energy estimates and the evaluation of secondary structure predictions, the largest maximal MFE_AD (18.4 kcal/mol in both) were obtained for the PairFold-Mathews method, while the minimal MFE_AD (13 kcal/mol for EVAL-FE and 11.88 kcal/mol for EVAL-SS) corresponds to the UNAFold method (see Methods for details). The average differences for the EVAL-FE methods range between 2.41 kcal/mol (UNAFold) and 3.16 kcal/mol (Vienna Package), while for the prediction methods the interval is slightly shifted towards zero. We also observed a similar improvement trend for MFE_AD standard deviations of EVAL-SS methods versus EVAL-FE methods, a phenomenon that can be explained by the intrinsic regression-based construction of the underlying DNA parameters used by each method.

**Table 1:** Summary of features for the data sets used in this study

Set	Num. duplexes	Seq. len.	*T *[C]	[*Na*]^+ ^[M]	Seq. conc. [M]
Aboul-ela et al. [32]	34	16	25, 50	1	[11e-6,440e-6]
Allawi et al.-1 [37]	24	9 - 12	37	1	1e-4
Allawi et al.-2 [20]	28	9 - 14	37	1	1e-4
Allawi et al.-3 [21]	22	9 - 14	37	1	1e-4
Bommarito et al. [43]	37	8 - 9	37	1	*n.r.*
Breslauer et al. [26]	12	6 - 16	25	1	*n.r.*
Clark et al. [44]	1	24	37	0.15	2.5e-6
Doktycz et al. [19]	140	8	25	1	2e-6
Gelfand et al. [45]	4	13	25	1	5e-5
LeBlanc et al. [46]	7	10 - 11	25	1	5e-5
Leonard et al. [22]	5	12	25	1	4e-4
Lesnik et al. [39]	14	8 - 21	37	0.1	4e-6
Li et al. [23]	12	8 - 10	25	1	6.1e-6
Nakano et al. [40]	21	6 - 14	37	0.1	8e-6
Petruska et al.-1 [47]	4	9	37	*n.r.*	*n.r.*
Petruska et al.-2 [36]	2	30	37	0.17	1e-4
Peyret et al. [48]	52	9 - 12	37	1	1e-4
Pirrung et al. [49]	2	25	25	0.1	1e-6
Plum et al. [50]	2	13	25	1	6e-6
Ratmeyer et al. [51]	2	12	37	1	6e-6
SantaLucia et al.-1 [29]	23	4 - 16	37	1	4e-4
SantaLucia et al.-2 [29]	10	12	24.85	1	5e-6
Sugimoto et al.-1 [30]	50	5 - 14	37	1	5e-6
Sugimoto et al.-2 [38]	1	8	37	*n.r.*	1e-4
Sugimoto et al.-3 [52]	8	6 - 8	37	1	*n.r.*
Tanaka et al. [34]	126	12 - 25	37	1	5e-5
Tibanyenda et al. [33]	3	16	24.85	1	17.5e-6
Wilson et al. [35]	3	11	25	0.4	*n.r.*
Wu et al. [53]	48	5 - 11	25, 37	1	1e-4
TOTAL:	695				

Each data set has the following characteristics: the number of sequence pairs (**Num. duplexes**), the length of the sequences (**Seq. len**), the experimental temperature measured in degrees Celsius for estimating free energies (**T**), the sodium concentration measured in molar units ([Na]^+^)and the sequence concentration (**Seq. conc**). The set of 695 DNA duplexes contains: (i) 143 perfect match free energies measured at a temperature of 37°*C *and a sodium concentration of 1 M, (ii) 197 perfect match duplexes measured at a temperature of 25°*C *and a sodium concentration of 1 M, (iii) 7 perfect match duplexes measured at a temperature of 50°*C *and a sodium concentration of 1 M, and (iv) 348 duplexes with mismatches measured at various temperatures and sodium concentrations. Note: **n.r**. denotes values that have not been reported in the original documents.

**Table 2:** Summary of results for free energy measurements obtained with EVAL-SS methods.

Method	Stats	MFE_AD [kcal/mol]	Pearson coeff. (r)		SSSI	Sens.	PPV	F-measure
MultiRNAFold	min	0.000	0.7565	4.35	40.00	0.1667	1	0.2857
(Mathews)	q1	0.340			100.00	1.0000	1	1.0000
	median	0.860			100.00	1.0000	1	1.0000
	mean	2.681			95.83	0.9547	1	0.9711
	q3	3.590			100.00	1.0000	1	1.0000
	max	18.400			100.00	1.0000	1	1.0000
	stddev	3.429			10.56	0.1224	0	0.09236
MultiRNAFold	min	0.000	0.7663	4.131	40.00	0.1667	1	0.2857
(SantaLucia)	q1	0.330			100.00	1.0000	1	1.0000
	median	0.720			100.00	1.0000	1	1.0000
	mean	2.528			96.44	0.9608	1	0.9747
	q3	3.510			100.00	1.0000	1	1.0000
	max	17.200			100.00	1.0000	1	1.0000
	stddev	3.269			10.23	0.1189	0	0.08966
	min	0.000	0.7660	3.992	40.00	0.1667	1	0.2857
	q1	0.256			100.00	1.0000	1	1.0000
	median	0.630			100.00	1.0000	1	1.0000
UNAFold	mean	2.374			96.08	0.9571	1	0.9724
	q3	3.016			100.00	1.0000	1	1.0000
	max	11.880			100.00	1.0000	1	1.0000
	stddev	3.212			10.66	0.1231	0	0.09234
Vienna	min	0.010	0.7630	3.667	5.882	0.0000	0.0000	0.0000
Package	q1	1.700			100.000	1.0000	1.0000	1.0000
	median	2.330			100.000	1.0000	1.0000	1.0000
	mean	3.025			95.210	0.9467	0.9856	0.9616
	q3	3.935			100.000	1.0000	1.0000	1.0000
	max	15.400			100.000	1.0000	1.0000	1.0000
	stddev	2.075			13.74	0.1581	0.1192	0.1387

Summary of results for free energy measurements obtained with EVAL-SS methods. The p-values for the Pearson correlation test were less than 2.2e-16 in all cases.

### Comparison of root mean squared errors (RMSE)

We measure the root mean squared error between experimentally determined and predicted free energies. In an ideal scenario where predicted values equal experimental values, the RMSE would be zero, thus the lower the RMSE value is, the closer the predicted values are to the experimental ones. Here, all methods produce comparably low RMSEs, the lowest EVAL-FE RMSE (3.876) and EVAL-SS RMSE (3.667) being obtained in both cases with Vienna Package (column 5 in Tables 3 and 4).

**Table 3:** Summary of results for free energy measurements obtained with EVAL-FE methods.

Method	Statistics	MFE_AD [kcal/mol]	Pearson coeff. (r)	RMSE
MultiRNAFold	min	0.0000	0.7352	4.418
(Mathews)	q1	0.300		
	median	0.800		
	mean	2.672		
	q3	3.395		
	max	18.400		
	stddev	3.521		
MultiRNAFold	min	0.0000	0.7456	4.223
(SantaLucia)	q1	0.330		
	median	0.680		
	mean	2.553		
	q3	3.390		
	max	17.200		
	stddev	3.367		
	min	0.0000	0.7434	4.101
	q1	0.2528		
	median	0.6128		
UNAFold	mean	2.4110		
	q3	2.9970		
	max	13.0000		
	stddev	3.319		
Vienna	min	0.0000	0.7413	3.876
Package	q1	1.820		
	median	2.440		
	mean	3.167		
	q3	3.965		
	max	15.400		
	stddev	2.236		

Summary of results for free energy measurements obtained with EVAL-FE methods. The p-values for the Pearson correlation test were less than 2.2e-16 in all cases.

**Table 4:** Estimated free energy parameters

ID	Doublet	[kcal/mol]	Counts	ID	Doublet	[kcal/mol]	Counts
1.	AA/TT	-0.838948	84	6.	CC/GG	-1.698997	74
2.	AC/TG	-1.394988	102	7.	CG/GC	-0.967002	106
3.	AG/TC	-1.323547	102	8.	GA/CT	-0.938327	101
4.	AT/TA	-0.375235	130	9.	GC/CG	-0.711466	126
5.	CA/GT	-1.406794	95	10.	TA/AT	-0.144092	136
ID	Triplet	[kcal/mol]	Counts	ID	Triplet	[kcal/mol]	Counts
1.	AAA/TTT	-0.844597	10	17.	CAG/GTC	-1.625284	23
2.	AAC/TTG	-1.841904	19	18.	CCA/GGT	-1.568813	18
3.	AAG/TTC	-1.201194	17	19.	CCC/GGG	-2.396507	17
4.	AAT/TTA	-0.991596	19	20.	CCG/GGC	-1.888906	22
5.	ACA/TGT	-1.121939	20	21.	CGA/GCT	-1.668273	19
6.	ACC/TGG	-1.793995	23	22.	CGC/GCG	-2.195726	23
7.	ACG/TGC	-1.615048	30	23.	CTA/GAT	-0.871636	40
8.	ACT/TGA	-0.781693	23	24.	CTC/GAG	-1.198450	16
9.	AGA/TCT	-1.103536	15	25.	GAA/CTT	-1.317278	18
10.	AGC/TCG	-1.528461	36	26.	GAC/CTG	-1.498999	29
11.	AGG/TCC	-1.323278	18	27.	GCA/CGT	-1.454430	21
12.	ATA/TAT	-0.562379	46	28.	GCC/CGG	-1.973081	24
13.	ATC/TAG	-1.157521	29	29.	GGA/CCT	-1.696158	20
14.	ATG/TAC	-1.263601	26	30.	GTA/CAT	-1.158422	32
15.	CAA/GTT	-0.988509	16	31.	TAA/ATT	-0.519499	27
16.	CAC/GTG	-2.088824	17	32.	TCA/AGT	-1.042342	19

Estimated free energy parameters for unique DNA NN doublets and triplets and their corresponding counts of appearance in the perfect match data set. All parameters have been estimated using experimental values measured at 37°*C *and 1 M sodium concentration.

### Comparison of Pearson correlation coefficients (r)

**Figure 1:**
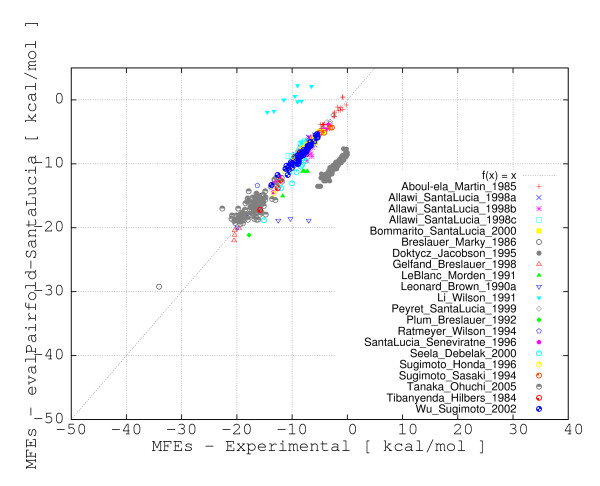
**Correlation plot for the evaluation of free energy estimates (EVAL-FE) obtained with MultiRNAFold (with SantaLucia parameters) versus experimental free energies**. The correlation of free energy estimates for all 695 DNA duplexes are represented. The plot depicts with different symbols and colors the source for each data point.

**Figure 2:**
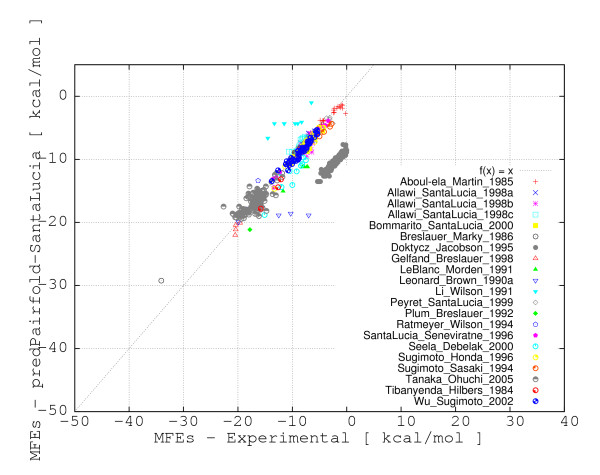
**Correlation plot for the evaluation of secondary structure predictions (EVAL-SS) obtained with MultiRNAFold (with SantaLucia parameters) versus experimental free energies**. The correlation of free energies for predicted secondary structures for all 695 DNA duplexes are represented. The plot depicts with different symbols and colors the source for each data point.

A correlation coefficient is traditionally defined as a symmetric, scale-invariant measure of association between two random variables, which takes values between -1 and 1. The extreme values indicate a perfect positive (1) or negative (-1) correlation, while 0 means no correlation. Positive Pearson Product Moment correlations are observed for all methods when experimental and evaluated or predicted free energies are considered as random variables. The highest Pearson correlation coefficients (~ .75 and ~ .77) are consistently obtained with the PairFold-SantaLucia method for both EVAL-FE and EVAL-SS, closely followed by UNAfold, Vienna Package and PairFold-Mathews. A major and consistent deviation from the correlation line of approximately 8 Kcal/mol for the data collected from Doktycz et al. [19] and a few other minor deviations for the data collected from four additional publications [20-23] were consistently noticed for all free energy calculation methods (see Figures 1 and 2). The majority of the deviations (e.g. Doktycz et al. [19]) may come from potentially different free energy interpolation functions used in those studies.

**Figure 3:**
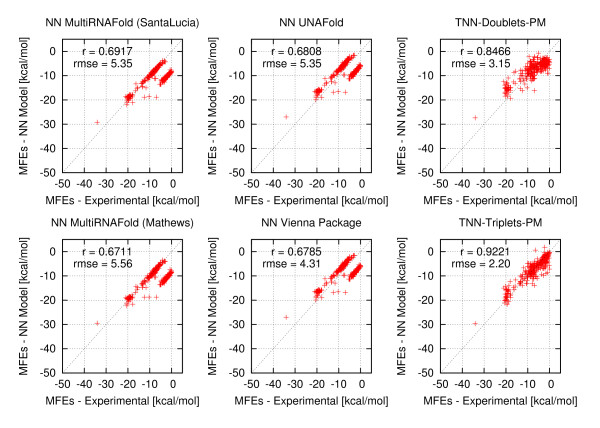
**Correlation plots for estimated versus experimental free energies of perfect matches**. Each correlation plot consists of 340 data points corresponding to all perfect match duplexes covering all temperatures, sequence and sodium concentrations. The top left plot depicts the correlation between experimental free energies and free energies estimated by MultiRNAFold with SantaLucia parameters. The Pearson correlation equals 0.6917 and the RMSE is 5.35. The bottom left plot depicts the correlation between experimental free energies and free energies estimated by MultiRNAFold with Mathews parameters. The Pearson correlation equals 0.6711 and the RMSE is 5.56. The top middle plot depicts the correlation between experimental free energies and free energies estimated by UNAFold. The Pearson correlation equals 0.6808 and the RMSE is 5.35. The bottom middle plot depicts the correlation between experimental free energies and free energies estimated by Vienna Package. The Pearson correlation equals 0.6785 and the RMSE is 4.31. The top right plot depicts the correlation between experimental free energies and free energies estimated by the TNN-Doublets-PM Model. The Pearson correlation equals 0.8466 and the RMSE is 3.15. The bottom right plot depicts the correlation between experimental free energies and free energies estimated by the TNN-Triplets-PM Model. The Pearson correlation equals 0.9221 and the RMSE is 2.20.

**Figure 4:**
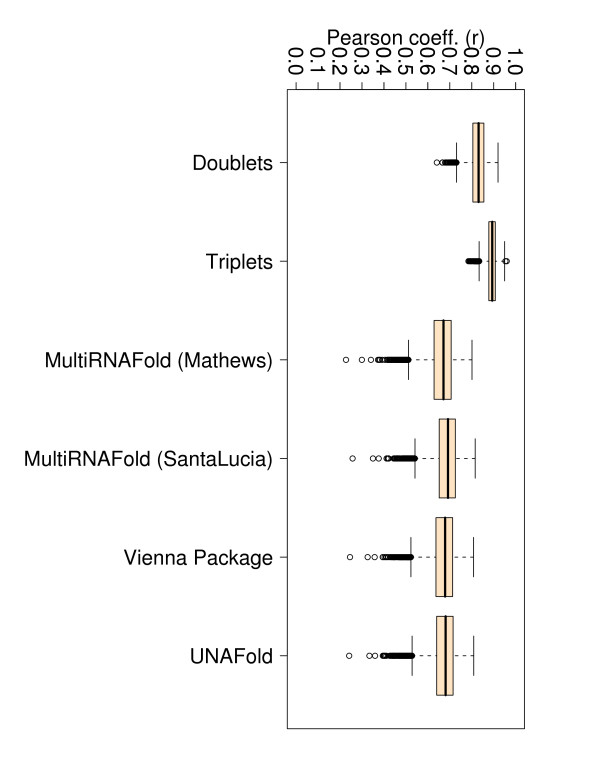
**Box plots for Pearson correlations (r) corresponding to all 340 perfect match duplexes**. The figure represents box plots for Pearson correlation coefficients for all 340 perfect match duplex free energies measured at various temperatures, sequence and sodium concentrations. The doublet- and triplet-based models were executed 10 000 times on randomly selected subsets with 67% training data and 33% testing data.

**Figure 5:**
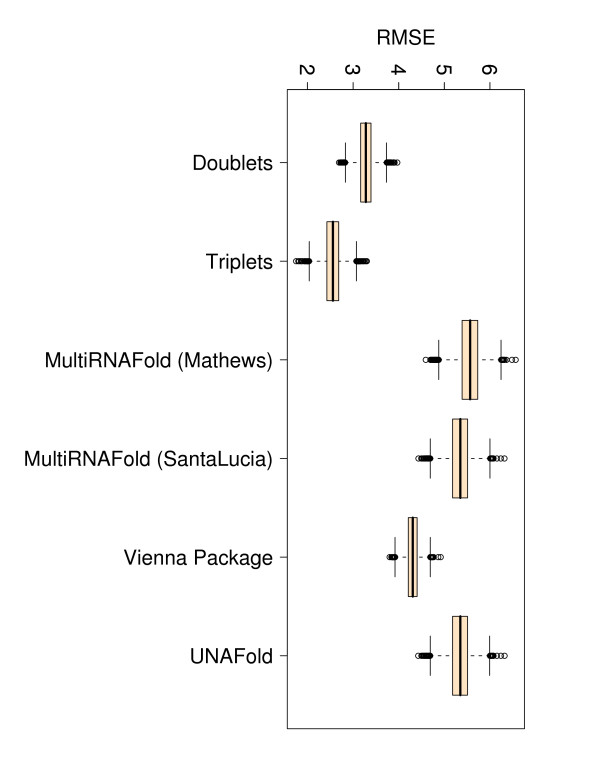
**Box plots for RMSEs corresponding to all 340 perfect match duplexes**. The figure represents box plots for RMSEs for all 340 perfect match duplex free energies measured at various temperatures, sequence and sodium concentrations. The doublet- and triplet-based models were executed 10 000 times on randomly selected subsets with 67% training data and 33% testing data.

**Figure 6:**
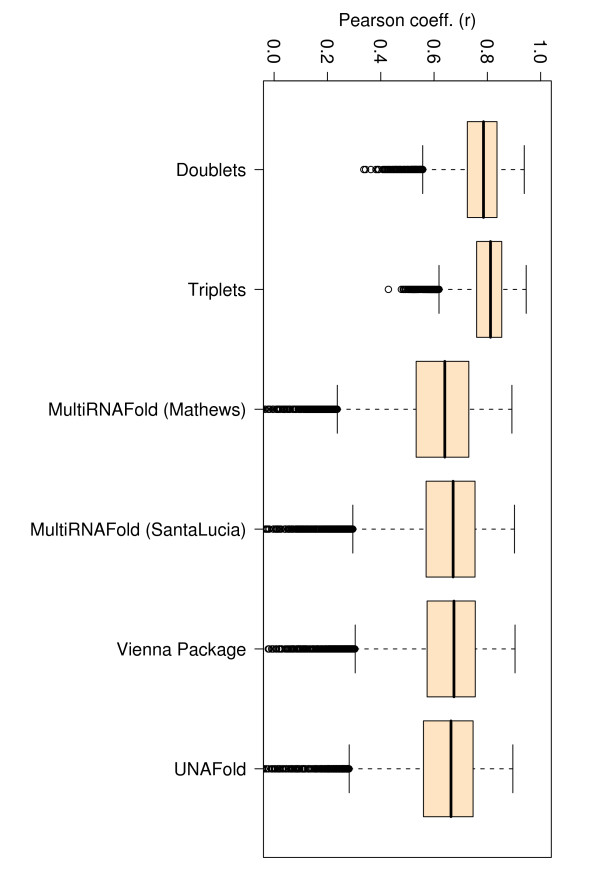
**Box plots for Pearson correlations (r) corresponding to 197 perfect match duplex free energies measured at 25°*C***. The figure represents box plots for Pearson correlation coefficients for 197 perfect match duplex free energies measured at 25°*C *and a sodium concentration of 1 M. The doublet- and triplet-based models were executed 10 000 times on randomly selected subsets with 67% training data and 33% testing data.

**Figure 7:**
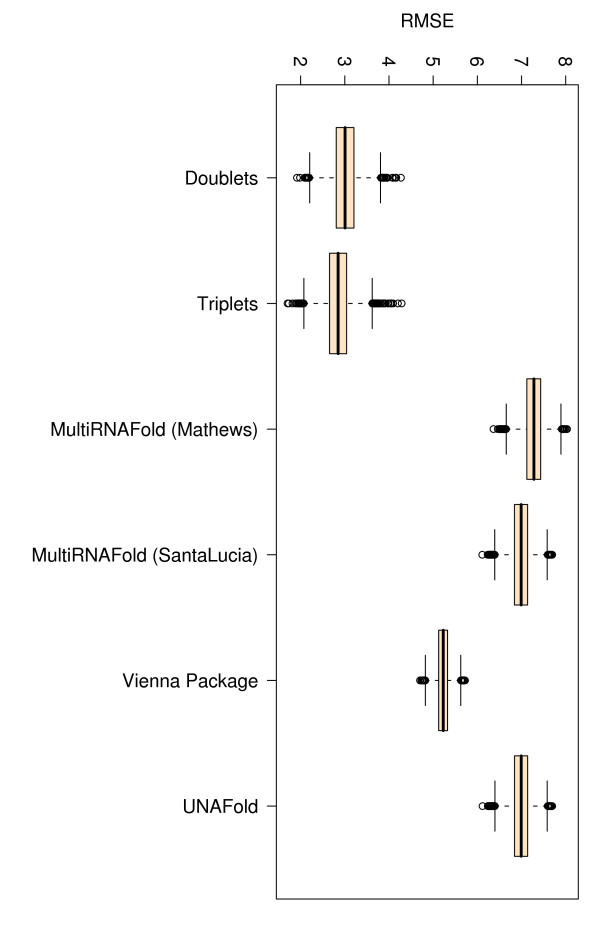
**Box plots for RMSEs corresponding to 197 perfect match duplex free energies measured at 25°*C***. The figure represents box plots for RMSEs for 197 perfect match duplex free energies measured at 25°*C *and a sodium concentration of 1 M. The doublet- and triplet-based models were executed 10 000 times on randomly selected subsets with 67% training data and 33% testing data.

**Figure 8:**
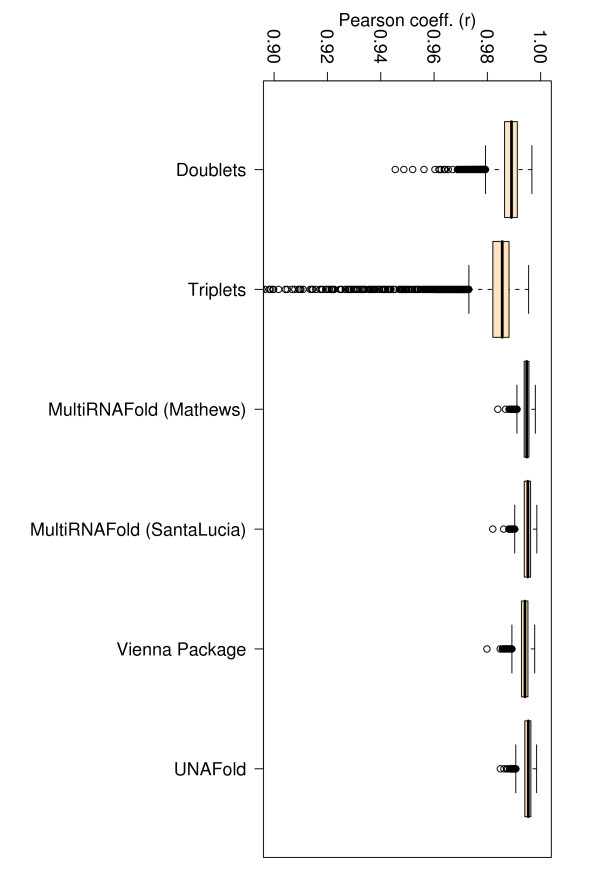
**Box plots for Pearson correlations (r) corresponding to 143 perfect match duplex free energies measured at 37°*C***. The figure represents box plots for Pearson correlation coefficients for 143 perfect match duplex free energies measured at 37°*C *and a sodium concentration of 1 M. The doublet- and triplet-based models were executed 10 000 times on randomly selected subsets with 67% training data and 33% testing data.

**Figure 9:**
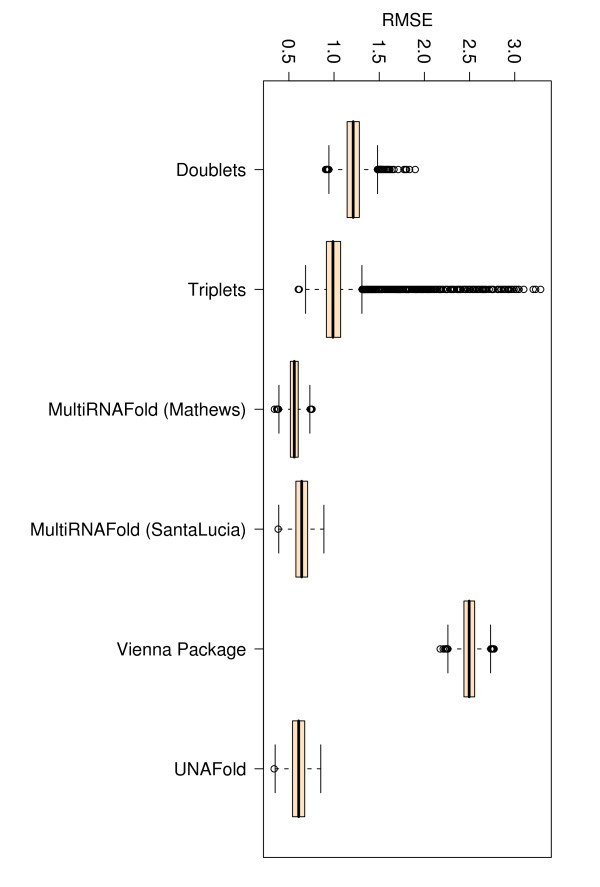
**Box plots for RMSEs corresponding to 143 perfect match duplex free energies measured at 37°*C***. The figure represents box plots for RMSEs for 143 perfect match duplex free energies measured at 37°*C *and a sodium concentration of 1 M. The doublet- and triplet-based models were executed 10 000 times on randomly selected subsets with 67% training data and 33% testing data.

If we consider only perfect match data, the TNN-Triplets-PM Model (see Methods) is capable of estimating free energies that correlate better (*r *= 0.92) with experimental values (see Figure 3), than all the other methods, which show an average correlation coefficient *r *= 0.68. We notice also an improvement in the RMSE for the TNN-Triplets-PM Model, compared to the other programs. To ensure that this improvement is due to the triplet aspect of the model rather than other confounding factors, we created a TNN-Doublets-PM Model that has been trained and evaluated on the same perfect match data set. A detailed description of the training and evaluation procedure is provided in Tables 5 and 6. For the complete data set with perfect matches measured at various temperatures and buffer concentrations, Figures 4, 5, 6, 7, 8 and 9 show that our TNN-Triplets-PM Model consistently produces better correlations and RMSEs, when we run a random design experiment using 10 000 randomly selected subsets with 67% duplexes (228 perfect match duplexes) used for training and 33% duplexes (112 perfect match duplexes) used for testing. The same high correlations can be observed when running the TNN-Triplets-PM Model on perfect match duplex free energies measured at a temperature of 25°*C *and 1 M sodium concentration, while for perfect match free energies measured at 37°*C *and 1 M sodium concentration, the other models produce better but still comparable correlations (0.9) and RMSEs (0.7) with the TNN-Triplets-PM Model.

**Table 5:** Model training

**Require: **A thermodynamic model *T*, an input set *S *with perfect match DNA duplexes.
**Ensure: **An optimal set of thermodynamic DNA parameters *X *for the input model
1: Initialize counts matrix *F *with zeros for all unique doublets/triplets
2: Initialize results matrix *R *with experimentally approximated free energies for each duplex
3: **for ***i *= 0 to ||*S*|| **do**
4: Count unique doublets/triplets in duplex *S*[*i*] and update *F*
5: **end for**
6: Solve the equation *X *= arg min_*X *_(*F *× *X *- *R*)^2^
7: **return ***X*

**Table 6:** Model evaluation

**Require: **A thermodynamic model *T*, an input set *S *with perfect match DNA duplexes.
**Ensure: **Vectors of Pearson correlations () and root mean square errors () for all duplexes.
1: Initialize correlations vector
2: Initialize root mean square errors vector
3: **for ***i *= 0 to 10 000 **do**
4: Training set *TrS *= 67% of randomly chosen data from *S*
5: Testing set *TeS *= remaining 33% of data from *S*
6: Train model *T *on data in *TrS*
7: Compute *r *and *RMSE *for each data point in *TeS*
8:
9:
10: **end for**
11: **return **vectors and

### Comparison of secondary structure similarity indexes of experimental and predicted secondary structures (SSSI)

The accuracy of secondary structure prediction for various methods can be evaluated by using the newly introduced measure described in equation 5. The SSSI measure simply calculates the percentage of correctly predicted secondary structure bonds corresponding to the positions in each secondary structure (corresponding to each sequence in the duplex) that match the position in the experimental secondary structure, normalized by the sum of sequence lengths. Comparable mean SSSI values were produced by all methods with a maximal value of 96.44% attained by PairFold-SantaLucia. The lowest value (95.21%) was obtained with Vienna Package (see column 6 in Table 2). All methods have large standard deviation for SSSI values, thus suggesting a wide sample distribution.

### Comparison of SENS, PPV and F for predicted secondary structures

**Figure 10:**
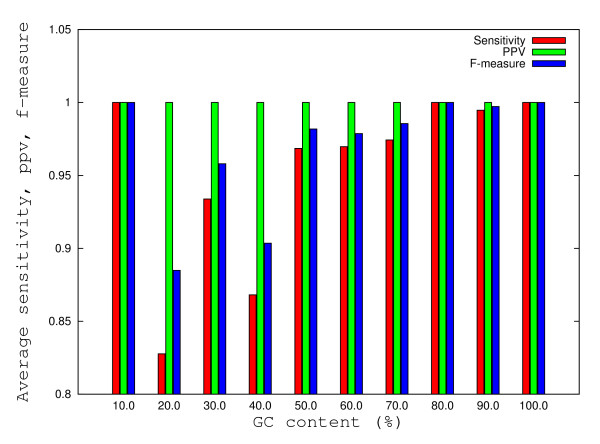
**Histograms of average variations of the SENS, PPV and F-measure with respect to sequence length**. The histograms corresponding to average variations of the SENS, PPV and F-measure (defined in Methods) with respect to sequence length were calculated for all 695 duplexes. All minimum free energies were calculated with PairFold-SantaLucia.

**Figure 11:**
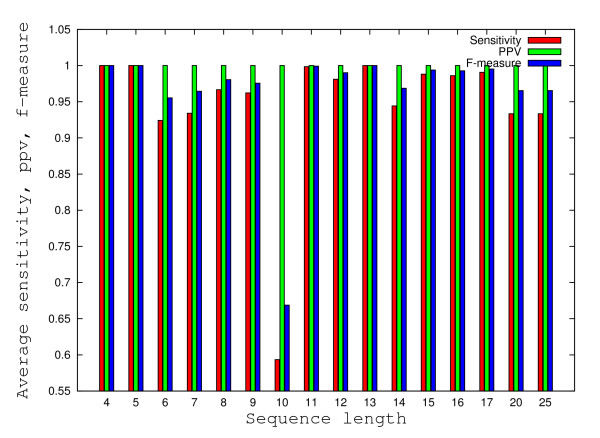
**Histograms of average variations of the SENS, PPV and F-measure with respect to GC-content percentage**. The histograms corresponding to average variations of the SENS, PPV and F-measure (defined in Methods) with respect to GC-content percentage were calculated for all 695 duplexes. All minimum free energies were calculated with PairFold-SantaLucia.

The analysis of the variation for sensitivities and F-measures with respect to sequence length and GC content percentages reveals a common pattern for all prediction methods. Mean sensitivities higher than 0.9 and mean F-measures higher than 0.95 were obtained for all methods and all sequence lengths with one exception. For sequences of length 10 a major drop in sensitivities and F-measures can be observed (see Figures 10 and 11). The main cause for the abrupt drop in sensitivities seem to apply mostly for sequences whose experimentally determined secondary structures contain two consecutive mismatches (collected from [23]), thus partially supporting the hypothesis that the prediction models under investigation seem to be optimized to produce better results for almost complementary pairs of DNA sequences. Next we look at how GC content % impacts the accuracy of prediction for the methods under consideration. While sensitivities and F-measures are higher than 0.9 for all methods for a wide range of GC content % intervals (e.g. 0% -10%, 40% - 100%), there are values for which sensitivities and F-measures drop under 0.9 for sequences with GC content percentages in the range 10% - 40%. While Pairfold-Mathews, Pairfold-SantaLucia and UNAFold generate predictions with sensitivities higher than 0.9 for sequences with GC content percentages in the range 20% - 30%, the Vienna Package has a mean sensitivity of only 0.8. For 3 out of 4 methods, the PPV equals 1 (maximum), while for the remaining one, namely the Vienna Package slightly lower mean values (0.98) were obtained.

### Comparison of free energy parameters for DNA doublets measured at 37°*C *and 1 M sodium concentration

**Figure 12:**
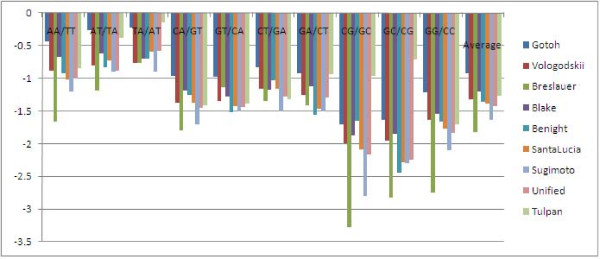
**Variation of doublet NN values for 9 sets of parameters**. Free energy values corresponding to nine sets (our set and 8 others) of thermodynamic nearest-neighbour doublet parameters at 37°*C *are displayed in this plot. Four (Gotoh, Vologodskii, Blake and Benight) out of the eight publicly available sets of doublet parameters correspond to models that do not account for initiation penalties for duplex formations [18], and the sodium concentration for their experiments was between 0.0195 M and 0.195 M. For the other 4 sets (Breslauer, SantaLucia, Sugimoto and Unified) the sodium concentration equals 1 M.

Table 2 presents the estimated free energy parameters for DNA doublets measured at 37°*C*. The set of 10 parameters corresponds to the best set obtained with the procedure explained in Table 6. We compared our set of NN free energy parameters at 37°*C *with eight other sets of parameters reported by SantaLucia [18], namely the sets obtained by Gotoh [24], Vologodskii [25], Breslauer [26], Blake [27], Benight [28], SantaLucia [29], Sugimoto [30] and the Unified set [31]. Our set of NN thermodynamic doublet parameters summarized in Figure 12 differs from the unified parameters by less than 0.5 kcal/mol in 8 out of 10 cases. We also notice that our NN set follows in general the reported qualitative trend in order of decreasing stability: GC/CG = CG/GC > GG/CC > CA/GT = GT/CA = GA/CT = CT/GA > AA/TT > AT/TA > TA/AT with one exception, namely GG/CC has a higher weight than GC/CG and CG/GC, an effect that could be caused by the low representation of the GG/CC doublets in the training set and by the absence of duplex initiation parameters in our model.

## Conclusions

In this work we showed that no major differences exist among free energy estimations of short DNA duplex hybridization when comparing four publicly available programs that employ various sets of thermodynamic parameters.

Here we introduce a simplified TNN Model based on triplets interactions for perfect match hybridizations of DNA duplexes. The model is able to approximate free energies for DNA duplexes under various experimental conditions with higher accuracy and lower RMSEs compared to the four publicly available programs considered in this work. The improvement is more noticeable for DNA duplexes at non-standard experimental temperature conditions (for example at 25°*C*). This improvement obtained with the TNN Model based on triplets could be explained by the presence of a larger set of parameters consisting of 32 unique triplets (compared to only 10 unique doublets in the classical TNN Model) that better capture the impact of sequence components on the overall free energy of a DNA duplex. An alternative and potential complementary explanation of these improvements is the use of a wider variety of experimental data points in the thermodynamic parameter extrapolation process (the model training stage) compared to the smaller and less diverse data sets used in the other four programs. Nevertheless, we notice that additional experimental data employing longer and more diverse sequences is required in order to obtain a better approximation of free energies for DNA duplexes at other non-standard experimental conditions.

Three extensions of the TNN-Triplets-PM Model might improve its performance, given that additional experimental data that covers a higher percentage of the parameters and experimental condition combinations is obtained experimentally: (i) the model can incorporate weighted additive terms that account for hybridization initialization, temperature, pH, sodium concentration or sequence concentrations; (ii) the model can incorporate symmetrical and asymmetrical internal loops, multi-branch loops, dangling ends and hairpin rules similar to those already existent in the classical TNN Model; (iii) the model can also incorporate positional dependencies of triplets with respect to the 5' and 3' ends of the sequences.

## Methods

The present study is divided into two major sections:

• **Evaluation of free energy estimates (EVAL-FE): **a comparative assessment of free energies calculated for DNA duplexes using different methods when both the duplex sequence and the duplex experimental secondary structure are given.

• **Evaluation of secondary structure predictions (EVAL-SS): **an accuracy assessment of secondary structure predictions when only the duplex sequence is given and the secondary structure is predicted.

### Data

**Figure 13:**
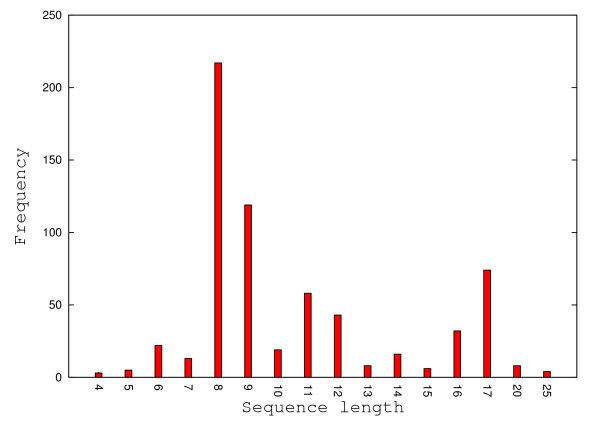
**The distribution of sequence lengths for the complete data set**. The sequence length distribution of 695 DNA duplexes. The 8-mers, 9-mers followed by 17-mers have the highest frequencies, while 4-mers, 5-mers and 25-mers have the lowest frequencies.

The benchmark data set used in this work consists of 695 experimental free energies and secondary structures for DNA duplexes, including 340 perfect matches and 355 imperfect matches. We collected these data from 29 publications and we present its characteristics in Table 1. We must mention that a total of 42 DNA duplexes were removed from the original data set (with 737 DNA duplexes - see Additional file 1) because the *ctEnergy *function from UNAFold failed to produce valid free energies, due to the lack of DNA parameters for mismatches. The removed data corresponds to 30 duplexes from [31], 4 duplexes from [32], 4 duplexes from [33], 2 duplexes from [34] and 2 duplexes from [35]. The lengths of DNA sequences in the data set range from 4 nucleotides [29] to 30 nucleotides [36], some of them (length 8 and 9) being over represented (see Figure 13).

**Figure 14:**
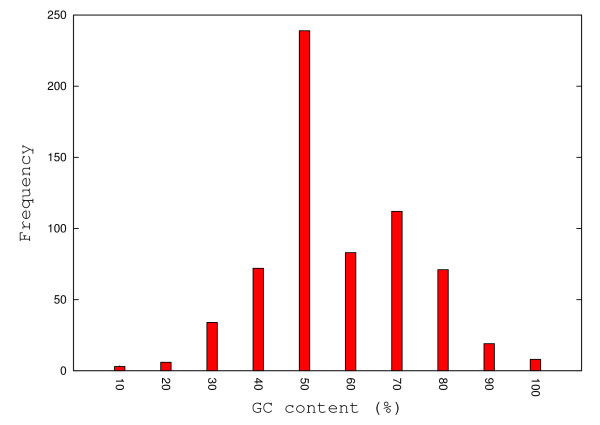
**The distribution of GC-content percentages for the complete data set**. The distribution of GC-content percentages for 695 duplexes. The majority of sequences have a 50% GC-content while only a few sequences have either low (10%, 20%) or high (100%) GC-content percentages.

The GC-content (%) of the sequences in the benchmark data set (see Figure 14) cover the whole spectra from 0% to 100%, with a dominant peak at 50%.

Sequence concentrations range from 17.5 × 10^-6 ^M in [33] to 10^-4 ^M in [20,21,31,37,38]. The sodium concentration varies from 0.1 M in [39] and [40] to 1 M in 20 out of 29 sources. The reported free energies were measured at reaction temperatures ranging between 24.85°*C *[33,41] and 50°*C *[32].

### Free energy calculations

In this study, three publicly available packages were used to calculate and compare the free energies for pairs of short DNA sequences: MultiRNAFold (with Mathews and SantaLucia parameters), UNAFold and the Vienna Package. All packages implement the TNN Model based on base doublet parameters.

The basic free energy calculations implemented in MultiRNAFold and Vienna Package are performed according to the Gibbs equation:(1)

where *G*° is the free energy measured, *H*° is the enthalpy, *T *is the absolute temperature measured in degrees Kelvin and *S*° is the entropy.

For a general two-state transition process of the type *A *+ *B *⇌ *AB *at equilibrium, the free energy change is calculated as follows:(2)

where *R *is the gas constant (1.98717 cal/(mol K)), *T *is the absolute temperature, and *k *is the equilibrium constant.

### Computational procedures

The two main sections of this study, namely the evaluation of free energy estimates and the evaluation of secondary structure predictions, employ computational procedures made available in the corresponding software packages. The evaluation of free energy estimates (**EVAL-FE**) includes the following procedures:

• The function *free energy pairfold (sequence1, sequence2, known structure) *is provided by the MultiRNAFold package to compute the free energy for two sequences when the known secondary structure is given. The *pairfold *wrapper has been slightly modified to accept as parameters: two sequences, the temperature, the set of parameters (Mathews or SantaLucia), the nucleic acid (DNA or RNA) and the type of hybridization (with or without intra-molecular interactions between nucleotides).

• The function *RNAeval *is provided by the Vienna Package to compute the free energy for two sequences when the known secondary structure is provided. We wrote a Python wrapper that calls this function with the following parameters: *-T temperature*, *-P dna.par*. The wrapper also pre-processes the sequence and structure input so to satisfy the interactivity requirements of the RNAeval function.

• The function *ctEnergy *is provided by the UNAFold Package to compute the free energy for two sequences when the known secondary structure is given. We wrote a Python wrapper that pre-processes the sequences and structures into a CT-formatted input file and calls the function with the following parameters: *-n DNA*, *-t temperature*, *-N sodium concentration*.

The evaluation of secondary structure predictions (**EVAL-SS**) includes the following procedures:

• The function *pairfold mfe (sequence1, sequence2, output structure) *is provided by the MultiRNAFold package to compute the minimum free energy secondary structure for two DNA sequences that fold into *'output structure'*. The *pairfold *wrapper has been slightly modified as described above.

• The function *RNAcofold *is provided by the Vienna Package to predict the free energy secondary structure for two sequences. A wrapper has been created for this function to accommodate the input and the parameters for the interactive interface as described above.

• The script *UNAFold.pl *is provided by the UNAFold Package to predict the free energy secondary structure for two sequences. We wrote a Python wrapper that pre-processes the sequences and structures into a CT-formatted input file and calls the function with the same parameters as for the *ctEnergy *function.

### The TNN-Triplets-PM Model

For the case when only free energies for perfect matches are evaluated, we explore an approach that extends the classical TNN Model by looking at base triplets. A similar approach was introduced in 1999 by [16]. For the classical TNN Model, only ten different nearest-neighbour interactions (out of 16) are possible for any Watson-Crick DNA duplex structure due to rotational identities. Here A is hydrogen bonded with T and G is hydrogen bonded with C. These interactions are AA/TT, AT/TA, TA/AT, CA/GT, GT/CA, CT/GA, GA/CT, CG/GC, GC/CG, and GG/CC. Here the slash, /, separates strands in anti parallel orientation (e.g., TC/AG means 5' - *TC *- 3' paired with 3' - *AG *- 5'). While the classical TNN model assumes that the stability of a DNA duplex depends on the identity and orientation of only close neighbouring base pairs, the one based on triplet interactions takes the approach one step further and assumes that the stability of a DNA duplex can be approximated if the first two neighbours of each base are considered. Since our goal is to examine and compare the impact of doublet versus triplet interactions on the accuracy of free energy estimations, the approach proposed in this paper relies solely on triplet interactions, while the one proposed by [16] uses a more complex cumulative approach that combines singlet, doublet and triplet interactions within the same model. Due to rotational identities, only 32 different nearest-neighbour interactions are possible (out of a total of 64) for any Watson-Crick triplet structure. These interactions are enumerated in Table 2 together with corresponding parametric values obtained via a least-mean squared optimization solution for equation 3.(3)

where *F *is a *N × *32 matrix of counts for all perfect match data points, *X *is a vector with 32 unknown triplet parameter values, and *R *is a vector with *N *free energy experimental values for perfect matches. We solve the following equation:(4)

These values were obtained by using an over determined system of *N *equations (3) and solving equation 4 with a least-mean squared optimization function (implemented in the backslash operator for matrices) available in Matlab 7.7. Here *N *takes the value 228 (67% of 340 perfect match free energies measured at 25°*C *and 37°*C*), 132 (67% from 197 perfect match free energies measured at 25°*C*), or 96 (67% of 145 perfect match free energies measured at 37°*C*). The system with *N *equations has been extrapolated by selecting from the initial data set only the free energy measurements for perfect match DNA duplexes and counting the frequency of triplets in each duplex. Thus, for each duplex, the sum of parametric values for each triplet multiplied with its counts equals the experimental free energy. While our model is very simple and currently does not take into consideration mismatches, internal loops, and dangling ends, its strength is given by its ability to estimate perfect match DNA duplex free energies for a wide range of sodium, sequence and target concentrations and temperatures. This strength is given by the presence of a large and mixed training data set that was used to extrapolate the nearest-neighbour (NN) parameters for both the doublet- and the triplet-based models.

### Model training and testing

The training process for the TNN-Triplets-PM Model is summarized in Table 5. We first process the input set, which contains perfect match DNA sequences and their corresponding experimentally derived free energies. The processing consists of scanning each perfect match sequence from left to right by moving a window of size 3 nucleotides (or 2 for the doublets) and counting the frequency of each of the 32 unique triplets. We record each frequency at corresponding positions (*i, j*) in matrix *F *and each experimentally derived free energy is recorded at position *i *in matrix *R*. Here *i *represents the number of the sequence in the set and *j *represents the number of the triplet (from 1 to 32), whose frequency is recorded. After matrices *F *and *R *have been populated, a solution for equation 4 is computed and the value of vector *X *containing free energy parameters for all the unique triplets is reported.

**Figure 15:**
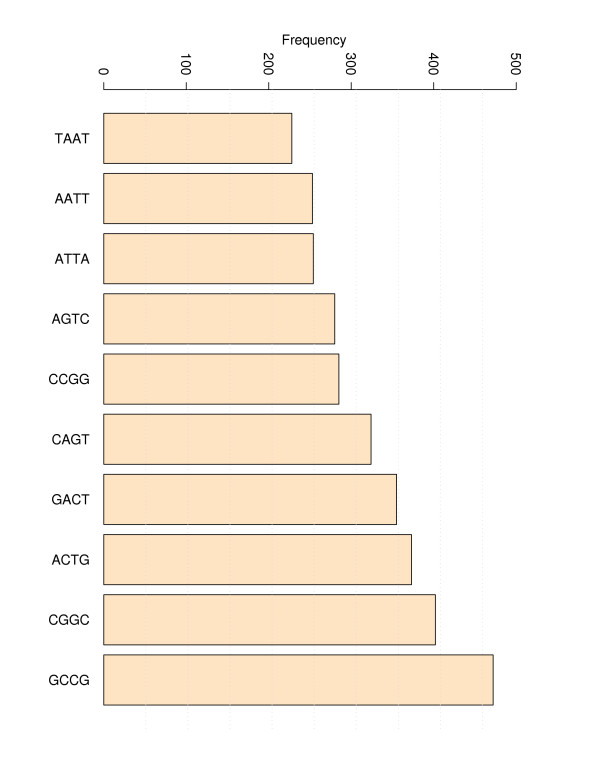
**Distribution of doublet frequencies**. The distribution of frequencies for all doublets corresponding to 340 perfect matches is presented. The doublet with the lowest frequency is TA/AT and the one with highest is GC/CG.

**Figure 16:**
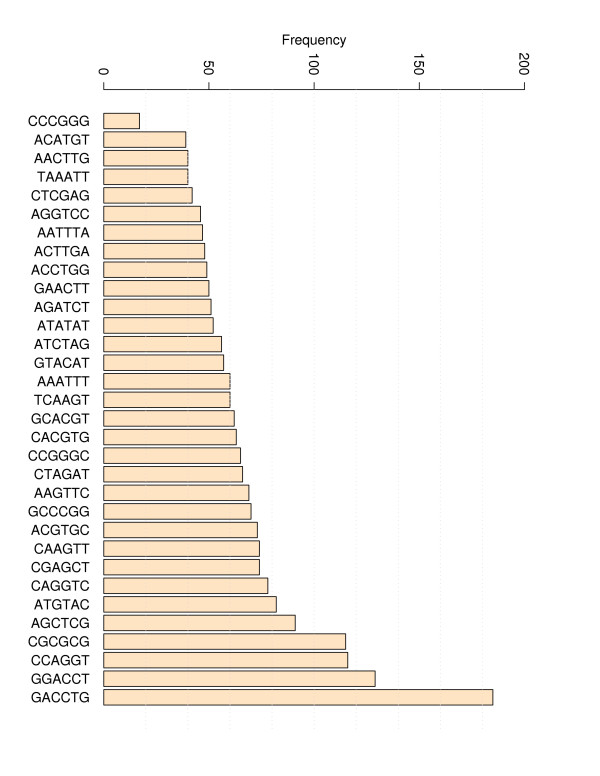
**Distribution of triplet frequencies**. The distribution of frequencies for all triplets corresponding to 340 perfect matches is presented. The triplet with the lowest frequency is CCC/GGG and the one with highest is GAC/CTG.

The evaluation process of the TNN-Triplets-PM Model is summarized in Table 6. The evaluation process is repeated 10 000 times in this work. Each iteration consists of the following steps. First the data set is divided uniformly at random in a training set, *TrS *consisting of 67% of the data and a testing set, *TeS *that contains the remaining 33%. Next, the training process described in Table 5 is used to extrapolate the first set of perfect match triplet parameters. The derived parameters are used next to compute the Pearson momentum correlation coefficients and the RMSEs for each DNA perfect match duplex from *TeS*. Each correlation coefficient and RMSE is recorded in corresponding vectors to be analyzed later. The complete coverage of the triplet space, i.e. all possible triplets during the generation of training and testing sets using a randomized mechanism is not ensured for some of the 10 000 sets mostly due to the presence of a few under-represented (less than 20 CCC/GGG) or over-represented (more than 180 GAC/CTG) triplets that characterize the data set with perfect matches (see Figures 15 and 16). Nevertheless, we noticed that the training sets that produced the best results cover completely the triplet space. The same coverage was observed for the doublets.

### Comparative measures

We use a large number of measures of similarity between experimental and computed free energies. Some of these measures were previously used by [42] to compare melting temperatures obtained with different methods and by [6] to estimate model parameters for RNA secondary structure prediction. If not stated otherwise, all comparisons in this paper were done on a data set comprising 695 pairs of DNA sequences collected from 29 publications. The measures used in this study are grouped in two categories, namely:

### Measures that evaluate accuracy of free energy estimations

The following measures are used for free energy estimations of the known structures, as well as free energy estimations of predicted structures.

• the observed absolute difference between experimental and estimated free energies (MFE_AD),

• the Pearson correlation coefficient (r),

• the root mean squared error (RMSE),

### Measures that evaluate accuracy of secondary structure predictions

• the secondary structure similarity index of experimental and predicted secondary structures (SSSI)

• the prediction sensitivity for secondary structures (SENS)

• the positive predictive value for secondary structures (PPV)

• the F-measure for predicted secondary structures (F)

For MFE_AD, SSSI, SENS, PPV and F we report the minimum, the first quartile, the median, the mean, the third quartile, the maximum and the standard deviation.

We define the secondary structure similarity index (**SSSI**) for two equally long structures as follows:(5)

where *s*1_*exp*_, *s*2_*exp *_are two equally long structures obtained experimentally, *s*1_*calc*_, *s*2_*calc *_are two equally long calculated structures, and *SS*(*a, b*) is the total number of identical characters at corresponding positions in both structures. *SSSI *represents the percentage of positions in which two structures agree.

Unlike similar measures that assign a +1 score for two identical base pairs in two duplex structures, SSSI assigns a +1 score for two base pairs that have either the start or the end positions identical. This mechanism allows the differentiation between duplex secondary structures that have either one (score +1) or both (score +2) bases in a base pair correctly predicted.

The sensitivity, positive predictive value and F-measure are defined as in [6], namely:(6)

### Computational infrastructure

The entire analysis of this study was done with R version 2.5.1, Perl 5.8.8 and Python 2.5. All computations were carried out on a Open SuSe 10.2 Linux (kernel version 2.6.18.2) machine equipped with a Pentium 4, 2.8 GHz processor with 1 GB of RAM.

## Authors' contributions

DT and MA planned the research, collected and curated the data, and wrote the paper. SL wrote the code for results computation and collection with UNAFold and the Vienna Package. DT wrote the code for results computation and collection with MultiRNAFold and analyzed the data. All authors read and approved the final manuscript.

## Supplementary Material

Additional file 1 **Data set in comma separated value**. The file contains information representing the data set used in this work. The data is structured on 15 columns as follows: (col 1) first sequence of the duplex, (col 2) second sequence of the duplex, (col 3) unique duplex ID containing the first and last authors of the papers that have first published the data, (col 4) dot-parenthesis notation of the secondary structure representation for the first sequence, (col 5) dot-parenthesis notation of the secondary structure representation for the second sequence, (col 6) experimental free energy measurement, (col 7) measurement error for the free energy, (col 8) experimental entropy measurement, (col 9) measurement error for the entropy, (col 10) experimental enthalpy measurement, (col 11) measurement error for the enthalpy, (col 12) experimental temperature of hybridization, (col 13) concentration for self-complementary sequences, (col 14) concentration for non self-complementary sequences, (col 15) [*N a*] ^+ ^concentration. Click here for file
